# Falciparum Malaria in Patient 9 Years after Leaving Malaria-Endemic Area 

**DOI:** 10.3201/eid1501.080909

**Published:** 2009-01

**Authors:** Caroline Theunissen, Peter Janssens, Anne Demulder, Denis Nouboussié, Marjan Van Esbroeck, Alfons Van Gompel, Jef Van den Ende

**Affiliations:** Brugmann University Hospital, Brussels, Belgium (C. Theunissen, P. Janssens, A. Demulder, D. Noubouossie); Prince Leopold Insitute of Tropical Medicine, Antwerp, Belgium (M. Van Esbroeck, A. Van Gompel, J. Van den Ende)

**Keywords:** Plasmodium falciparum malaria, suitcase malaria, late malaria, Belgium, Guinea-Conakry, letter

**To the Editor:** A 30-year-old African man, without any specific medical history, came to the emergency department of Brugmann University Hospital in Brussels, Belgium, on March 18, 2008, because of malaise, profuse transpiration, and dizziness that day. He also reported a 3-day history of muscle pain and pain while urinating, for which his general practitioner prescribed ciprofloxacin. Originally from Guinea-Conakry, the patient reported no travel outside Belgium after his arrival >9 years earlier. No recurrent fever episodes were noted during this period. Two weeks before becoming ill, a friend visiting from Guinea-Conakry stayed at his home for 7 days.

Results of a physical examination were normal, except for a temperature of 37.5°C. Blood analysis showed moderate anemia (hemoglobin 12.9 g/dL) and thrombocytopenia (platelet count 73,000/μL) with total bilirubin and C-reactive protein levels of 1.5 mg/dL and 7.0 mg/dL, respectively. Because we suspected subfebrile malaria in this patient, a blood smear was prepared. It showed ring-shaped trophozoites of *Plasmodium falciparum* with a parasite density of 0.1%. Serologic tests showed an antibody titer to *Plasmodium* spp. of 3,200. A blood sample was sent to the national reference laboratory at the Institute of Tropical Medicine in Antwerp. The diagnosis of *P*. *falciparum* malaria was confirmed by microscopy and real-time PCR. Follow-up was uneventful because the patient responded to a 7-day course of oral quinine and doxycycline.

Malaria, a potential life-threatening disease caused by *P*. *falciparum*, usually occurs within 2 months after the bite of an infective mosquito. A few reports mention a delay of >1 year between exposure and initial clinical symptoms, probably related to the disappearance of residual protective immunity in immigrants (*1*,*2*). Impaired immunity has also been linked to late malaria, implicating a chronic low-grade *P*. *falciparum* infection that becomes clinically evident in an immunocompromised person (*3*). In addition, several cases of malaria without any travel history to a malaria-endemic region have been described. A possible explanation for this type of malaria is exposure to an imported *Anopheles* spp. mosquito, referred to as airport, luggage, or container malaria (*4*). Transmission by indigenous anopheline mosquitoes when weather conditions are favorable has been reported in some European countries (*5*,*6*). Cases of *P*. *falciparum* malaria without any evidence of a mosquito bite have been reported and related to transfusion of parasitized erythrocytes, intravenous drug use, or accidental needlestick injuries (*4*).

We report a clinically atypical case of late *P*. *falciparum* malaria that may have been contracted by the bite of an anopheline mosquito captured in the luggage of the patient’s visiting friend (*7*). Unreported travel to a malaria-endemic region was possible but unlikely because our patient stayed illegally in Belgium and leaving the country would risk being repatriated to Guinea-Conakry. Indigenous malaria was excluded because he became ill during the winter, a time when proliferation of local *Anopheles* spp. in Belgium is difficult. The patient did not receive any recent blood transfusions and denied being an intravenous drug user, although this possibility cannot be excluded.

This type of malaria, also known as luggage or suitcase malaria, makes adequate and timely diagnosis difficult because a history of exposure to a possibly malaria-infected mosquito is apparently absent. Moreover, our patient had few classic symptoms or signs, such as high-grade fever, chills, or headaches; this pattern complicates diagnosis. This lack of typical malaria symptoms may be related to the fact that before coming to the hospital, the patient took ciprofloxacin, which has in vitro activity against *P*. *falciparum*. Another possible reason is residual immunity to malaria, which was no longer protective but still capable of attenuating symptoms or signs of malaria. This finding implicates recrudescence of disease after a long period of asymptomatic infection with *P*. *falciparum*. Serologic analysis detected high levels of antibodies to *Plasmodium* spp., which suggests a chronic infection rather than a new one.

It is generally accepted that protective immunity wanes after several months of nonexposure. Support for this thesis is the frequency of clinical malaria in African adults who visit their families after a long stay in a country where the disease is not endemic. However, these cases might be caused by antigenic variation of *P*. *falciparum* in the area visited, which would enable the parasite to evade the host’s immune response (*8*). Residual immunologic memory against *P*. *falciparum* has been suggested, which would link persistent immunity with late recrudescence or with less severe or complicated disease in immigrants (*2*,*9*). Moreover, *P*. *falciparum* has been transmitted through blood transfusions from donors from malaria-endemic regions several years after exposure, which suggests long subpatent periods (*10*).

This case highlights the problem of diagnosing *P*. *falciparum* malaria in patients without a recent travel history to malaria-endemic areas. In such cases, autochthonous malaria, whether transmitted by an imported or an indigenous mosquito or by infected blood cells or needles, should be excluded. Residual protective immunity, even after several years of nonexposure to *P*. *falciparum*, may explain persistent asymptomatic infection and late recrudescence of disease.
